# Self-assessment of quality of life in patients after suffering from aneurysmal subarachnoid hemorrhage, principal component analysis

**DOI:** 10.1038/s41598-025-11523-8

**Published:** 2025-07-16

**Authors:** Sergio A. Calero-Martinez, Nazeer Aboud, Georg Simion, Ricardo Borda, Daniel Sarmiento, Marcel Kamp, Christian Senft, Nazife Dinc

**Affiliations:** 1 Department of Neurosurgery, Jena University Hospital, Friedrich-Schiller-University Jena, Jena, Germany; 2 Department of Statistics, El Bosque University, Bogota, Colombia; 3 Center for Palliative and Neuro-palliative Care, Brandenburg Medical School, Theodor Fontane, Faculty of Heath Sciences Brandenburg, Rüdersdorf Berlin, Germany

**Keywords:** Aneurysmal subarachnoid hemorrhage (aSAH), WHO QoL questionnaire, Patient-Reported outcomes, Endovascular treatment, Microsurgical clipping, Principal component analysis, Neuro-vascular interactions, Social neuroscience, Medical research

## Abstract

Quality of life (QoL) is one of the parameters that characterize the treatment success after aneurysmal subarachnoid hemorrhage, along with overall survival and independent living without the need for care. Thus, the main aim of this research was to evaluate the quality of life of patients after endovascular or microsurgical treatment of ruptured cerebral aneurysms. This study was performed at the Jena University Hospital in Germany. A total of 32 patients completed the German version of the WHO QoL Questionnaire during routine follow-up visits at our neurovascular outpatient clinic between January and September 2024. Analyses were stratified by the type of treatment. Patients who underwent endovascular treatment have better subjective reports of QoL in the psychological and environment domains, whereas those who underwent microsurgical clipping showed better outcomes in physical and social domains. Aneurysm location did not significantly impact QoL. To conclude, these findings suggest that different treatment modalities influence distinct aspects of QoL. Implementing treatment-tailored strategies, alongside comprehensive management of subarachnoid hemorrhage-related risks, may further enhance recovery and optimize functional outcomes.

**Supplementary Information:**

The online version contains supplementary material available at 10.1038/s41598-025-11523-8.

## Introduction

The Quality of Life (QoL) in patients who experience an aneurysmal subarachnoid hemorrhage (aSAH), is often significantly impacted due to the severity of the condition and its complications. Aneurysm rupture frequently results in acute neurological damage, leading to long-term physical, cognitive, and emotional impairments^1,2^.

Patients with aSAH have a reported fatality between 8.3 and 66.7%. Nieuwkamp et al. reported that 55% of patients regain independent function, 19% remain dependent, and 26% die^3^. Survivors may face a spectrum of challenges ranging from mobility issues and chronic pain to memory deficits and reduced ability to perform daily activities. This marked decline in functional independence often translates to a decreased overall QoL, even in cases where medical intervention is successful^4,5^.

Anxiety, depression, and post-traumatic stress disorder (PTSD) are common in individuals recovering from an aneurysm rupture. Studies have found that 41% of patients experience depression and/or PTSD symptoms 7 months post-aSAH^6^, while 32% meet PTSD diagnostic criteria^7^. Anxiety levels remain high and stable for up to 2 years after aSAH, with 59% of patients showing clinically significant anxiety^8^. These psychological issues are associated with poorer outcomes, including higher unemployment rates 6 months post-aSAH^9^. These mental health issues can stem from the trauma of the event, the uncertainty surrounding recovery, or feelings of dependency on caregivers. Moreover, cognitive impairments, including difficulty with concentration and decision-making, further exacerbate emotional distress, complicating reintegration into social and professional environments^10^.

The aim of surgical or endovascular treatment is to reduce the risk of bleeding, prevent progression, and maintain neurological functioning. The survival and QoL of patients with ruptured aneurysms, despite the advanced treatments, is limited^3,5^; thus, there is ongoing interest in assessing the impacts of treatment on patient’s QoL. These findings highlight the importance of identifying and addressing psychological morbidity in aSAH survivors, even in those patients with good physical recovery, to improve their long-term outcomes and QoL. In this study, we reported the QoL outcomes from patients treated in our clinic, weather with endovascular coiling or microsurgical clipping. Dependency was revisited in every follow up after the treatment using the full ordinal range of the modified Rankin Scale (mRS). We estimated and compared the overall effectiveness of the treatments regarding the quality of life of the treated patients reported by themselves or their legal companion, using the WHOQOL-questionary.

## Patients and methods

### Population

**Fig. 1:**
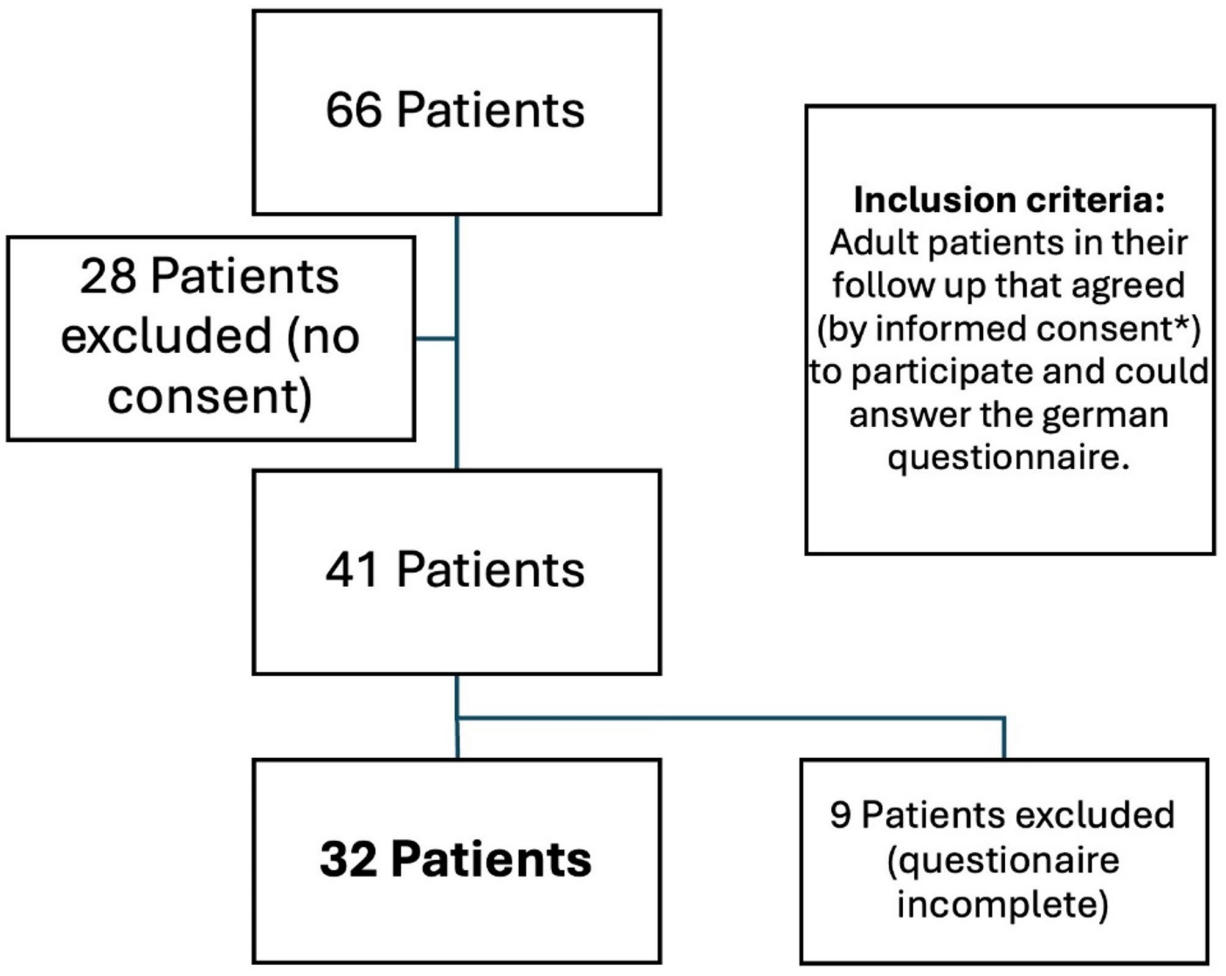
Flowchart of included and excluded patients. Selection process of patients who attended their regular follow-ups at the vascular neurosurgical outpatient clinic of the University Hospital Jena between January and September 2024 after experiencing aneurysmal subarachnoid hemorrhage. Initially, 63 patients were considered for inclusion; however, only 32 met the eligibility criteria. 9 patients were excluded because of missing data in the questionnaire. * Informed consent by themselves or their legal companion.

We prospectively selected patients from January to September 2024, who had suffered from aneurysmal SAH and were attending to their regular follow-up appointments one year after the event at the neurovascular outpatient clinic at the University Hospital of Jena, Department of Neurosurgery. Only patients that consented to participate in this study by themselves or through their legal accompanies and who were able to complete the German questionnaire independently or with the assistance of their legal guardian as well as those who responded to at least 80% of the QoL questionnaires were included in the study. The demographic and clinical data were retrospectively collected. In the flow chart are described the included and excluded patients (Fig. 1). In Table 1 are described the demographic variables of the patients treated with coiling or clipping. Table 2 resumes the clinical features, whereas Table 3 describes the scores related to QoL.

**Table 1:** Patient characteristics.

Variables	Clip (*n* = 13)	Coil (*n* = 19)
Sex (m: f)	6:7	4:15
Age (years old)	48,1 (23–68)	48,3 (28–71)
height (cm)	172,5 (158–188)	166,8 (155–192)
weight (Kg)	81,95 (43–107)	75,11 (50–105)
BMI	27 (16,3–34,9)	26,8 (17,5–41)
living situation		
1. home	4 (30,8)	6 (31,6)
2. apartment	5 (38,5)	9 (47,4)
3. assisted living	0	0
4. nursing home	0	0
5. living alone	1 (7,7)	1 (5,3)
6. living with partner / family	3 (23,1)	3 (15,8)
7. other	0	0
marital status		
1. single	4 (30,8)	2 (10,5)
2. married	6 (46,2)	11 (57,9)
3. divorced	0	2 (10,5)
4. widowed	3 (23,1)	3 (15,8)
5. other	0	0
children	11 (84,6)	15 (78,9)
parents	7 (53,8)	11 (57,9)
siblings	12 (92,3)	13 (68,4)
friends	13 (100)	18 (94,7)
university degree	2 (15,4)	6 (31,6)
job	5 (38,5)	8 (41,1)
Need for care	5 (38,5)	4 (21,1)

Descriptive characteristics of patients stratified by treatment. n = number of patients; values in parentheses represent the minimum and maximum range or percentage.

**Table 2:** Clinical features.

Variables	Clip (*n* = 13)	Coil (*n* = 19)
Aneurysm Location		
ICA	2 (15,4)	3 (15,8)
ACA	6 (46,2)	10 (52,6)
MCA	4 (30,8)	1 (5,3)
PCA	0	1 (5,3)
Basilar	1(7,7)	4 (21,1)
Hunt and Hess		
HH 1	5 (38,5)	3 (15,8)
HH 2	3 (23,1)	10 (52,6)
HH 3	1 (7,7)	2 (10,5)
HH 4	3 (23,1)	0
HH 5	5 (38,5)	4 (21,1)
Modified Fisher Scale		
F1	0	1 (5,3)
F2	2 (15,4)	4 (21,1)
F3	2 (15,4)	7 (36,8)
F4	9 (69,2)	7 (36,8)
WFNS		
WFNS 1	6 (46,2)	8 (42,1)
WFNS 2	3 (23,1)	5 (26,3)
WFNS 3	1 (7,7)	1 (5,3)
WFNS 4	1 (7,7)	1 (5,3)
WFNS 5	2 (15,4)	4 (21,1)
Shunt	5 (38,5)	6 (31,6)
Craniectomy	5 (38,5)	0
mRS at patient’s discharged		
mRS 0	0	0
mRS 1	5 (38,5)	4 (21,1)
mRS 2	2 (15,4)	8 (41,1)
mRS 3	1 (7,7)	5 (26,3)
mRS 4	3 (23,1)	1 (5,3)
mRS 5	2 (15,4)	1 (5,3)
mRS 6	0	0
mRS after one year		
mRS 0	3 (23,1)	7 (36,8)
mRS 1	5 (38,5)	8 (41,1)
mRS 2	0	2 (10,5)
mRS 3	4 (30,8)	1 (5,3)
mRS 4	1 (7,7)	0
mRS 5	0	0
mRS 6	0	0

Descriptive table of the clinical features of the included patients stratified by treatment. n: number of patients, values in parentheses represent the percentage.

**Table 3:** QoL scores and clinical scales.

Domain	Scale Grade	Hunt Hess (*n*)		Fisher (*n*)		WFNS (*n*)	
Physical	1	8	*0*,026*	1	*0*,*765*	14	*0*,*074*
	2	14		6		8	
	3	2		9		2	
	4	3		18		2	
	5	5				6	
Social	1	8	*0*,*267*	1	*0*,*670*	14	*0*,*309*
	2	14		6		8	
	3	2		9		2	
	4	3		18		2	
	5	5				6	
Psychological	1	8	*0*,*820*	1	*0*,*612*	14	*0*,*248*
	2	14		6		8	
	3	2		9		2	
	4	3		18		2	
	5	5				6	
Environmental	1	8	*0*,*059*	1	*0*,*739*	14	*0*,*133*
	2	14		6		8	
	3	2		9		2	
	4	3		18		2	
	5	5				6	
Total	1	8	*0*,*155*	1	*0*,*805*	14	*0*,*230*
	2	14		6		8	
	3	2		9		2	
	4	3		18		2	
	5	5				6	

Comparison between QoL and clinical scales scores of the included patients stratified by QoL-Domains. ANOVA, *p-value ≤ 0.05, CI 95%.

### QoL questionary

Our study was based on the WHO Quality of Life questionnaire (WHOQOL, German version)^11,12^. This questionnaire includes items that assess various aspects or domains of quality of life: physical, social, psychological, and environmental. The patients completed the questionnaire only once during their routine follow-up visits.

**Table 4:** QoL scores vs. type of treatment.

	Clip (*n* = 13)	Coil (*n* = 19)	*p*-value
Total	65,13 (43,34–84,15)	65,81 (25–84)	0.883
Domains			
Physical Health	53,29 (28,57–78,57)	51,31 (10,71–71,43)	0.714
Psychological Health	64,61 (29,16–91,66)	66,34 (16,67–87,50)	0.782
Social relations	69,23 (41,66–87,50)	68,42 (33,33–100)	0.889
Environment	73,38 (50–93,75)	77,18 (39,29–93,75)	0.467

Comparison between QoL-Scores and type of treatment of the included patients stratified by QoL-Domains. t-test, *p-value ≤ 0.05, CI 95%.

**Table 5:** QoL scores vs. mRS at discharge.

		Sum of Squares	df	Mean Square	F	Sig.
Physical health score	Between Groups	1871,62	4	467,905	2,545	0,06
	Within Groups	4780,838	26	183,878		
	Total	6652,458	30			
Psychological health score	Between Groups	1775,801	4	443,95	1,659	0,19
	Within Groups	6957,677	26	267,603		
	Total	8733,479	30			
Social relations score	Between Groups	504,477	4	126,119	0,482	0,75
	Within Groups	6806,231	26	261,778		
	Total	7310,708	30			
Enviroment score	Between Groups	1901,131	4	475,283	2,894	0,04*
	Within Groups	4270,508	26	164,25		
	Total	6171,639	30			
QoL Total Score	Between Groups	1171,424	4	292,856	2,13	0,11
	Within Groups	3574,074	26	137,464		
	Total	4745,497	30			

Comparison between QoL and mRS scores at discharged. ANOVA with post hoc analysis, *p-value ≤ 0.05, CI 95%.

**Table 6:** QoL scores vs. mRS at 12 months.

		Sum of Squares	df	Mean Square	F	Sig.
Physical health score	Between Groups	2330,026	3	776,675	4,57	0,011*
	Within Groups	4078,381	24	169,933		
	Total	6408,407	27			
Psychological health score	Between Groups	1954,759	3	651,586	2,432	0,09
	Within Groups	6430,081	24	267,92		
	Total	838,484	27			
Social relations score	Between Groups	1102,666	3	367,555	1,524	0,234
	Within Groups	5787,215	24	241,134		
	Total	6889,881	27			
Enviroment score	Between Groups	1509,324	3	503,108	2,737	0,066
	Within Groups	4412,242	24	183,843		
	Total	5921,566	27			
QoL Total Score	Between Groups	1355,302	3	451,767	3,323	0,037*
	Within Groups	3262,741	24	135,948		
	Total	4618,043	27			

Comparison between QoL and mRS scores 12 months after the bleeding event. ANOVA with post hoc analysis, *p-value ≤ 0.05, CI 95%.

### Statistical analysis

We stratified between patients treated endovascularly and surgically. Pearson`s correlation coefficient was calculated for all variables to stablish bivariate relationships. We used parametric or non-parametric tests as needed to assess differences between the variables and QOL results, as well as the treatment groups. Categorical variables were compared using Chi-square tests. A Wilcoxon signed-rank test was performed to compare variables with non-normal distribution, according to the goodness-of-fit test. ANOVA tests were used for the comparison of categorical variables. Two tailed p-values were considered statistically significant at ≤ 0.05.

**Fig. 2:**
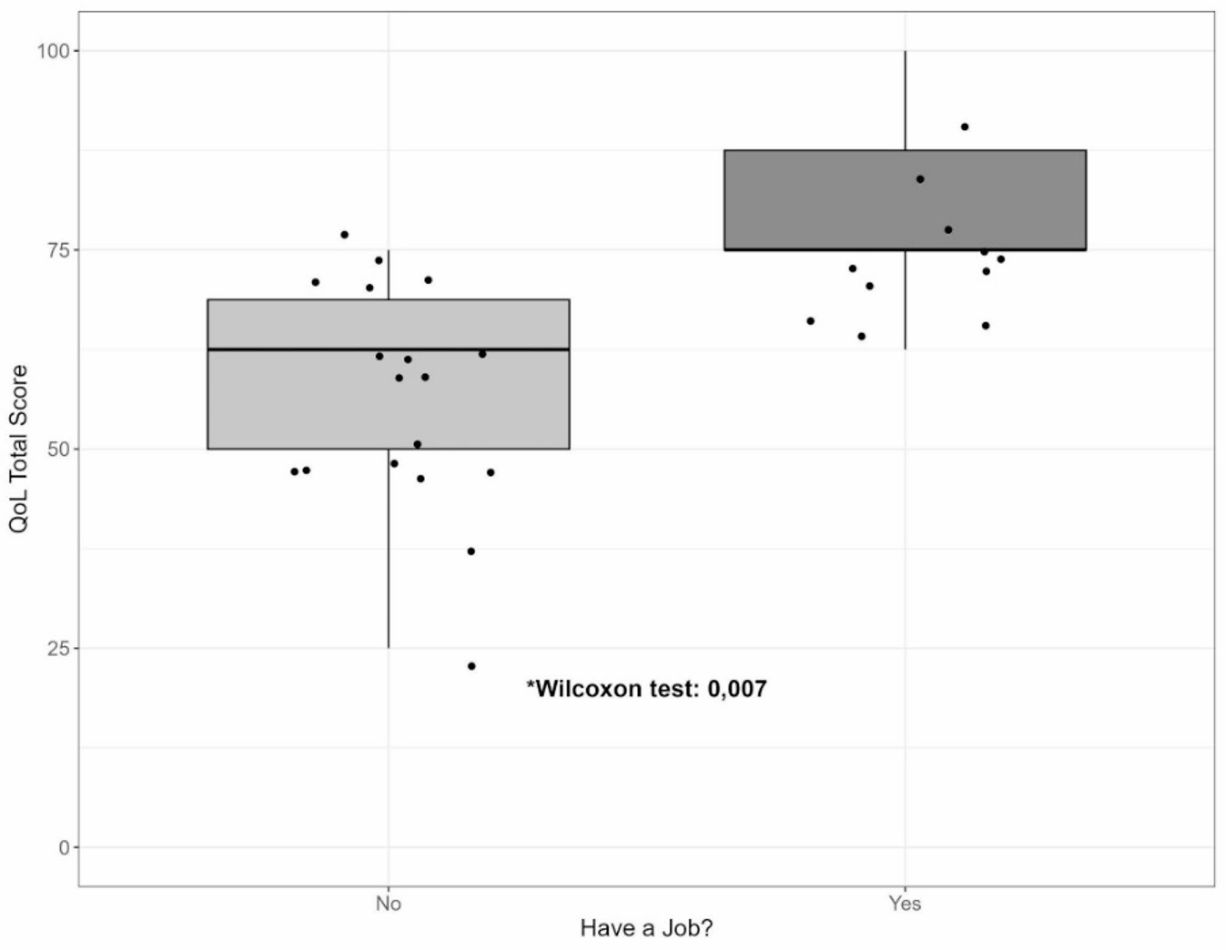
Box plot illustrating the association between current employment status (x-axis) and perceived quality of life (y-axis). A Wilcoxon test was conducted to assess statistical significance, revealing a significant difference (p-value < 0.05).

**Fig. 3:**
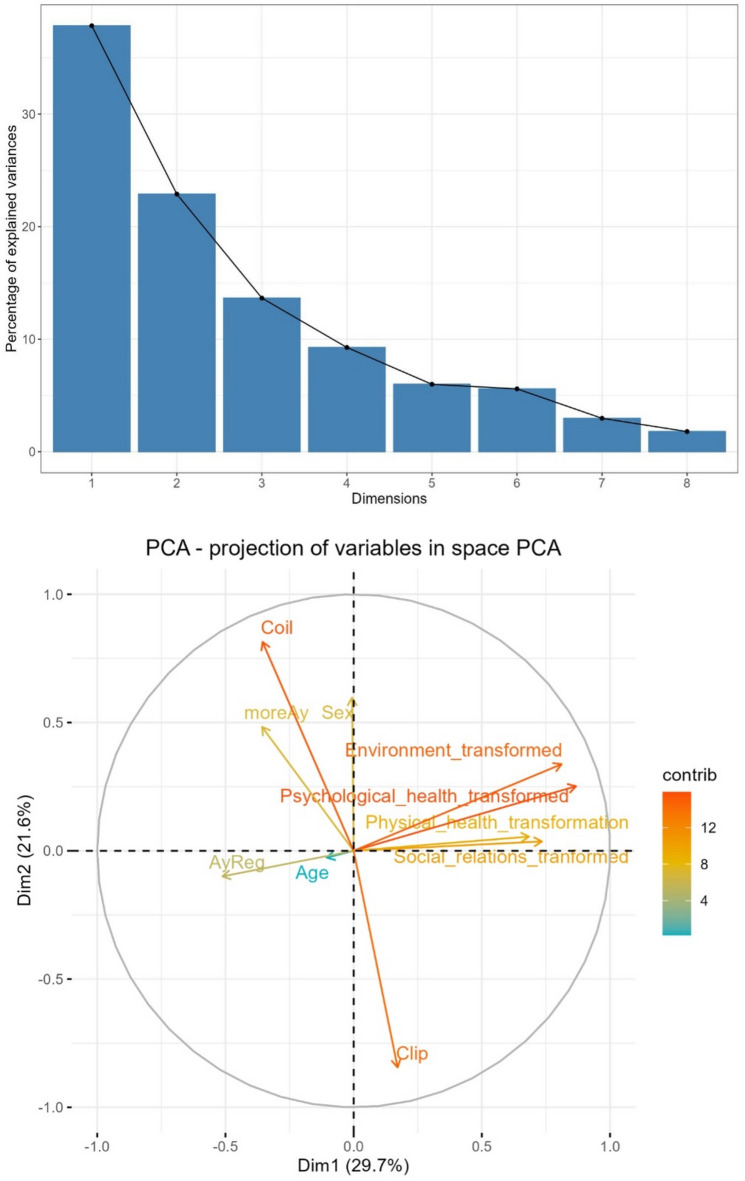
Principal component analysis. Scree Plot (Top): Each bar represents the percentage of variance explained by a principal component. The curve helps identify the optimal number of components to retain, typically by locating the “elbow” (here after dimension 5) where the variance gain diminishes. Biplot (Bottom): Arrows indicate how much each variable contributes (Contrib) to the principal components. Longer arrows and more intense colors highlight variables with greater influence on the orientation of the axes. Variables that are close together (< 90°) and have similar directions are positively correlated, while those that diverge and form a large angle (close to 180°) are negative correlated or independent.

Due to the relatively small sample size in relation to a larger number of potential influencing variables, we used PCA primarily to identify underlying patterns and dimensionality within the data. Using PCA, the optimal number of components for the analysis was determined. In this analysis, we simultaneously evaluated the variables that could influence the patients’ QoL reports, namely age, sex, presence of multiple brain aneurysms, aneurysm location. Hence, considering the total explained variance criterion, two components were identified to explain 51.34% of the total variance. Based on the acceleration factor, the inflection point was reached with these two components, while parallel analysis suggested retaining four components (Fig. 3; Table 8, Supplementary Table 1). A second PCA was performed including also the Hunt and Hess clinical scale (Supplementary Table and Fig. 2).

**Table 7:** QoL scores vs. having a job.

	having a job?	*N*	Mean	*p*-value
**Physical health score**	no	19	45,11	< 0,001*
	yes	13	62,36	
**Psychological health score**	no	19	60,55	0,037*
	yes	13	73,08	
**Social relations score**	no	19	66,67	0,374
	yes	13	71,79	
**Enviroment score**	no	19	70,48	0,011*
	yes	13	83,17	
**QoL Total Score**	no	19	60,70	0,006*
	yes	13	72,60	

Comparison between QoL scores and the employment status of the patients. Wilcoxon test, *p-value ≤ 0.05, CI 95%.

The results are reported in accordance with the STROBE guideline for epidemiological studies^13^.

The statistical analysis was performed using SPSS (IBM Corp. Released 2023. IBM SPSS Statistics for Windows, Version 29.0.2.0 Armonk, NY: IBM Corp) and R software (R Core Team, 2021. R: A language and environment for statistical computing. R Foundation for Statistical Computing, Vienna, Austria. URL https://www.R-project.org/).

### Standard protocol approvals, registrations, and patient consents

The study was conducted in accordance with the Helsinki declaration. We obtained local ethical committee approval for the study before enrolling patients. The study number was 2022–2744. Appropriate informed consent was obtained from all subjects and/or their legal guardian(s). No images requiring consent for publication are used in this manuscript.

## Results

### Demographics

We analyzed the questionnaires and clinical characteristics of 32 patients (Fig. 1; Table 1): 13 were (40.6%) treated endovascularly and 19 (59.4%) surgically. The average ages were 48.1 and 48.3 (23–60 and 28–71 years old), respectively. Most of the patients were female (68,75%). None of the demographic variables demonstrated a statistically significant correlation with QoL scores (Supplementary Fig. 1). Only 9 Patients reported need for care, 5 in the coil and 4 in the clipping group, no significant differences were found between patients requiring care after the aneurysmal rupture and treatment. The need for care did not show a statistically significant correlation with QoL scores (Supplementary Fig. 1).

The level of education did not differ significantly between the groups, nor was it associated with variations in QoL scores. Furthermore, having friends or family members alive such as children, parents, or siblings did not correlate with higher QoL scores.

Reporting current employment during follow-ups showed positive significant differences in quality of life (Fig. 2). Every domain other than “Social relations” showed significant differences when the patients have a job compared to the unemployed patients (p-value are described in Table 7).

**Table 8:** PCA Results.

Component	Eigenvalue	% of Variance	Cumulative Variance (%)
1	2,97467484	29,7467484	29,7467484
2	2,15961939	21,5961939	51,3429423
3	1,32388518	13,2388518	64,5817941
**4**	**1**,**09808539**	**10**,**9808539**	**75**,**5626481**
5	0,91587338	9,15873383	84,7213819
6	0,57508614	5,75086139	90,4722433
7	0,43907573	4,39075732	94,8630006
8	0,23397332	2,33973325	97,2027338
9	0,15846866	1,5846866	98,7874204
10	0,12125796	1,21257956	100

Principal Component Analysis (PCA) Results. The PCA method explains over 75% of the total variance after the fourth component, providing insight into the distribution of QoL domains and the total score across treatment groups.

### Clinical features

Variables related to aneurysmal location, shunting or craniectomy, as well as the clinical scales are described in Table 2. The location of the aneurysm did not correlate with a subjective perception of poor or good QoL. Among clinical scores after aneurysmal rupture, only patients exhibiting a Hunt and Hess (H&H) grade 5 demonstrated better QoL outcomes in the domain related to physical health (p-value 0,026, Table 3). After Post Hoc analysis, patients reporting a H&H 5 compared to those reporting a H&H 2 showed statistically significances regarding QoL. Remarkably, patients who had a H&H 5 have better QoL reports. More deeply, we found that 5 (15,6%) patients have had a H&H 5, those patients also reported better mRS assessments after 12 months. Here, the mean age of this participant was 39 years old, and 4 of them corresponded to females. Furthermore, we found that whether WFNS nor Fisher Scales correlate with QoL (p-values in Table 3). Additional treatments, such as shunting or craniectomy, also had no impact on the QoL of patients treated with coiling or clipping after a ruptured brain aneurysm. Regarding type of treatment and QoL, we did not find statistically significant differences between the groups (Table 4). Furthermore, we found statistically significant differences between mRS assessments and some of the QoL domains (p-values listed in Tables 5 and 6).

### PCA and QoL

The best cumulative variance for the PCA was achieved after the fourth component (75.56%, Table 8). Patients treated with clipping showed a positive correlation with the physical and social quality of life domains (variance explained by PC1: 29.74%), whereas those treated with coiling reported higher quality of life scores in the psychological and environmental domains (variance explained by PC2: 21.59%).

As shown in Fig. 3, the patients were grouped into two dimensions: coiling and clipping. We found that coiling was positively correlated with being female and had good reports for QoL, specifically in the psychological and environmental domains. In contrast, clipping showed a positive correlation with physical and social QoL. The location of the aneurysm and age did not correlate with the QoL domains. In a second round of analysis, we incorporated the H&H scale into the PCA; however, we did not observe any significant alterations compared to the previously identified correlations (Supplementary Figure and Table 2).

## Discussion

The study suggests after PCA analysis that coiling correlated with higher psychological and environmental QoL, whereas clipping was associated with better physical and social QoL. One possible explanation for these differences may lie in the recovery trajectory after each procedure. Microsurgical clipping is more invasive and typically requires longer hospitalization and structured rehabilitation, which could support physical and social reintegration. Endovascular coiling, on the other hand, may allow earlier discharge and quicker psychological adjustment. Employment status was strongly associated with better QoL. H&H grade 5 was unexpectedly linked to better QoL, while WFNS and Fisher scales showed no correlation. These findings highlight the multifaceted nature of post-aSAH recovery and the role of vocational reintegration in improving QoL.

The QoL in patients who experience an aneurysm rupture, is often significantly impacted due to the severity of the condition and its complications^2,10^. Aneurysm rupture frequently results in acute neurological damage, leading to long-term physical, cognitive, and emotional impairments. Survivors may face a spectrum of challenges ranging from mobility issues and chronic pain to memory deficits and reduced ability to perform daily activities^2^. This marked decline in functional independence often translates to a decreased overall QoL, even in cases where medical intervention is successful^14^. Moreover, cognitive impairments, including difficulty with concentration and decision-making, further exacerbate emotional distress, complicating reintegration into social and professional environments^10^.

Age is a critical determinant; older patients tend to experience worse clinical outcomes after aSAH due to reduced neuroplasticity and comorbidities^15^. Studies show that patients over 65 years often have poorer functional recovery and higher mortality rates after SAH compared to younger individuals^15,16^. In our study we did not observe any significance relation between age and QoL or treatment. We also assessed other variables, for instance BMI, living situation and marital status. However, we did not find any correlations between them and QoL.

Clinical scales used to assess the severity of aSAH show varying correlations with patient outcomes and QoL. The Glasgow Coma Scale (GCS) demonstrates the strongest correlation with QoL, while the Fisher score shows the weakest^17^. According with the literature, all three scales - H&H, GCS, and Fisher - correlate with discharge modified Rankin Scale (mRS) scores, with GCS being the most predictive of outcomes^18^. However, even patients classified as having “good outcomes” often experience reduced QoL in both physical and psychosocial domains^19^. Radiological scales, such as the modified Fisher scale, may be better predictors of delayed cerebral ischemia than clinical scales^20^. We found no associations between WFNS and Fisher with QoL reports. Nonetheless, in our analysis, patients with a higher H&H reported better QoL, this could be correlated with the fact that those patients recovered almost completely after one year. Although these results appear counterintuitive, the H&H scale may capture broader systemic and neurological impairment than WFNS, potentially explaining this discrepancy. However, the limited sample size warrants cautious interpretation. Other factors that could be associated to these findings are related to age and gender, the last, also proposed as an important variable in the principal component analysis.

While some studies found no association between QoL and mRS scores^21^, others reported correlations between these measures^22^. Long-term survivors may experience QoL improvements over time, even a decade after aSAH^23^. However, patients classified as having “good outcomes” (mRS 0–2) still reported low health-related QoL scores^24^. After reviewing our data, we found that patients with a good mRS exhibited better QoL.

Remarkably, our research indicates that having a job correlates positively with overall QoL after aSAH, these findings correspond with the literature. Several studies showed that 56–61% of previously employed patients return to work after aSAH^25,26^. Returning to work is associated with higher life satisfaction scores and better QoL^26,27^. However, many survivors face challenges in work productivity due to neuropsychological deficits, particularly in executive function, psychomotor speed, and memory^28,29^. Despite these difficulties, long-term follow-up studies reveal that general QoL scores for aSAH survivors are within the normal range, even 20–28 years post-hemorrhage^26^. These findings emphasize the importance of vocational reintegration and rehabilitation in improving overall QoL for aSAH survivors.

The limitations of our study, beyond the retrospective analyze, remain in the limited number of patients. First, the sample size is relatively small, as only 32 patients out of 69 met the inclusion criteria. This limited number may affect the generalizability of our findings to the broader population of patients with aneurysmal subarachnoid hemorrhage (aSAH). As an example, we saw the absence of associations between QoL and aneurysm location that may be due to the limited sample size and anatomical heterogeneity. For instance, anterior communicating artery aneurysms are known to affect cognitive domains, yet this was not reflected in our findings. Additionally, the requirement that patients be able to complete a German-language questionnaire may have introduced a selection bias, excluding non-German speakers or those with cognitive impairments that prevented them from fully understanding or responding to the questionnaire. Furthermore, the study included only patients who were in follow-up and agreed to participate. This voluntary participation may have led to selection bias, as patients with more severe neurological impairments or less favorable outcomes may have been less likely to participate. Similarly, patients who required a legal guardian to provide consent may represent a different subset of the aSAH population in terms of severity and recovery. Nevertheless, the study compensates with the robust data and variables, stratifying better the issues surrounding QoL after aSAH.

To our knowledge, this is the first study that evaluates together the QoL of patients with aneurysmal rupture and their demographics, clinical and paraclinical scales, social and private relationships, patient environment as well as endovascular and surgical treatment.

Treatment-tailored strategies, along with addressing subarachnoid hemorrhage-related risks, can significantly enhance recovery and functional outcomes. Family involvement and social support networks are equally important in fostering a sense of security and belonging during recovery. In addition, newer therapeutic approaches, including neuroplasticity-enhancing interventions and personalized rehabilitation strategies, hold promise in improving functional outcomes and QoL for these patients^30^. Comprehensive, individualized care that integrates physical, cognitive, and emotional support is essential to mitigate the long-term impacts of aSAH on QoL. Future research should explore interventions that account for these variables to optimize patient recovery and QoL.

## Conclusions

In conclusion, our findings suggest that QoL after aSAH is multifactorial and extends beyond physical recovery. While functional status (mRS) aligns with better QoL, specific domains such as psychological and environmental well-being appear to differ by treatment strategy. Employment status was a key determinant of higher QoL. These findings highlight the need for individualized, multidimensional post-aSAH care strategies. Future prospective studies with larger cohorts are warranted to validate these associations and guide tailored interventions. Future efforts should individualize patient´s necessities. Comprehensive care, increased awareness of the psychological and social implications of aneurysm rupture, and continued research into innovative treatment modalities should be taken care. By addressing these challenges, the long-term QoL of patients can be substantially improved, offering them a better chance at leading fulfilling lives post-recovery. However, most robust studies must be held to confirm our results.

## Electronic supplementary material

Below is the link to the electronic supplementary material.

Supplementary Material 1

## Data Availability

The dataset is not publicly available due to conditions of the ethics approval. Data on a cohort level may be made available by the corresponding author upon reasonable request.
