# Supplementary material for: Self-assessment of quality of life in patients after suffering from aneurysmal subarachnoid hemorrhage, principal component analysis
Source: Sci Rep. 2025 Jul 16;15:25674. doi: 10.1038/s41598-025-11523-8 (PMC12263825; doi:10.1038/s41598-025-11523-8)
Supplement: Supplementary file 1 — Supplementary Material 1 [file 41598_2025_11523_MOESM1_ESM.docx]

Supp. Figure 1. correlations

Figure 1. Correlations. Variables with positive correlations are highlighted in yellow, while those with negative correlations are shown in red. Pearson Correlation Test was used. A two-tailed P-value < 0.05 was considered statistically significant.

Supp. Table 1. PCA

| ***Eigenvalues*** | | | | | | | |
| --- | --- | --- | --- | --- | --- | --- | --- |
|  | Dim 1 | Dim 2 | Dim 3 | Dim 4 | Dim 5 | Dim 6 | |
| Variance. | 2.975 | 2.160 | 1.324 | 1.098 | 0.916 | 0.575 | |
| % of var. | 29.747 | 21.596 | 13.239 | 10.981 | 9.159 | 5.751 | |
| Cumulative % of var. | 29.747 | 51.343 | 65.582 | 75.563 | 84.721 | 90.472 | |
| ***Variables*** | | | | | | | |
| Sex | -0.006 | 0.001 | 0.000 | 0.597 | 16.513 | 0.357 | |
| Age | -0.105 | 0.370 | 0.001 | -0.027 | 0.033 | 0.001 | |
| AyReg | -0.512 | 8.804 | 0.262 | -0.100 | 0.461 | 0.010 | |
| MoreAy | -0.357 | 4.295 | 0.128 | 0.483 | 10.818 | 0.234 | |
| Coil | -0.356 | 4.249 | 0.126 | 0.815 | 30.731 | 0.664 | |
| Clip | 0.171 | 0.989 | 0.029 | -0.845 | 33.024 | 0.713 | |
| Psychological Health | 0.868 | 25.302 | 0.753 | 0.252 | 2.936 | 0.063 | |
| Social Relations | 0.735 | 18.142 | 0.540 | 0.036 | 0.062 | 0.001 | |
| Environment | 0.811 | 22.114 | 0.658 | 0.338 | 5.280 | 0.114 | |
| Physical Health | 0.684 | 15.735 | 0.468 | 0.055 | 0.142 | 0.003 | |
| ***Individuals (3 first)*** | | | | | | | |
|  | Dist | Dim 1 | Ctr | Cos2 | Dim 2 | Ctr | Cos |
| 1 | 2.811 | -0.067 | 0.005 | 0.001 | 1.838 | 4.890 | 0.428 |
| 2 | 3.382 | 1.224 | 1.573 | 0.131 | 0.111 | 0.018 | 0.001 |
| 3 | 3.107 | 1.986 | 4.143 | 0.408 | -0.941 | 1.281 | 0.092 |

Supp. Table 1. Eigenvalues and variables used for Principal Component Analysis (PCA). AyReg: vascular location of the aneurysm. MoreAy: more than one aneurysm.

Suppl. Figure 2. PCA 2


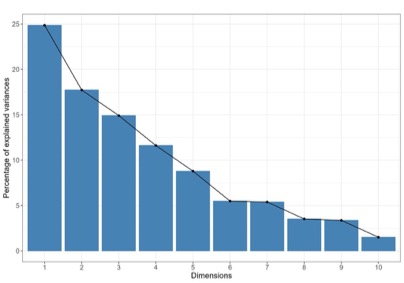


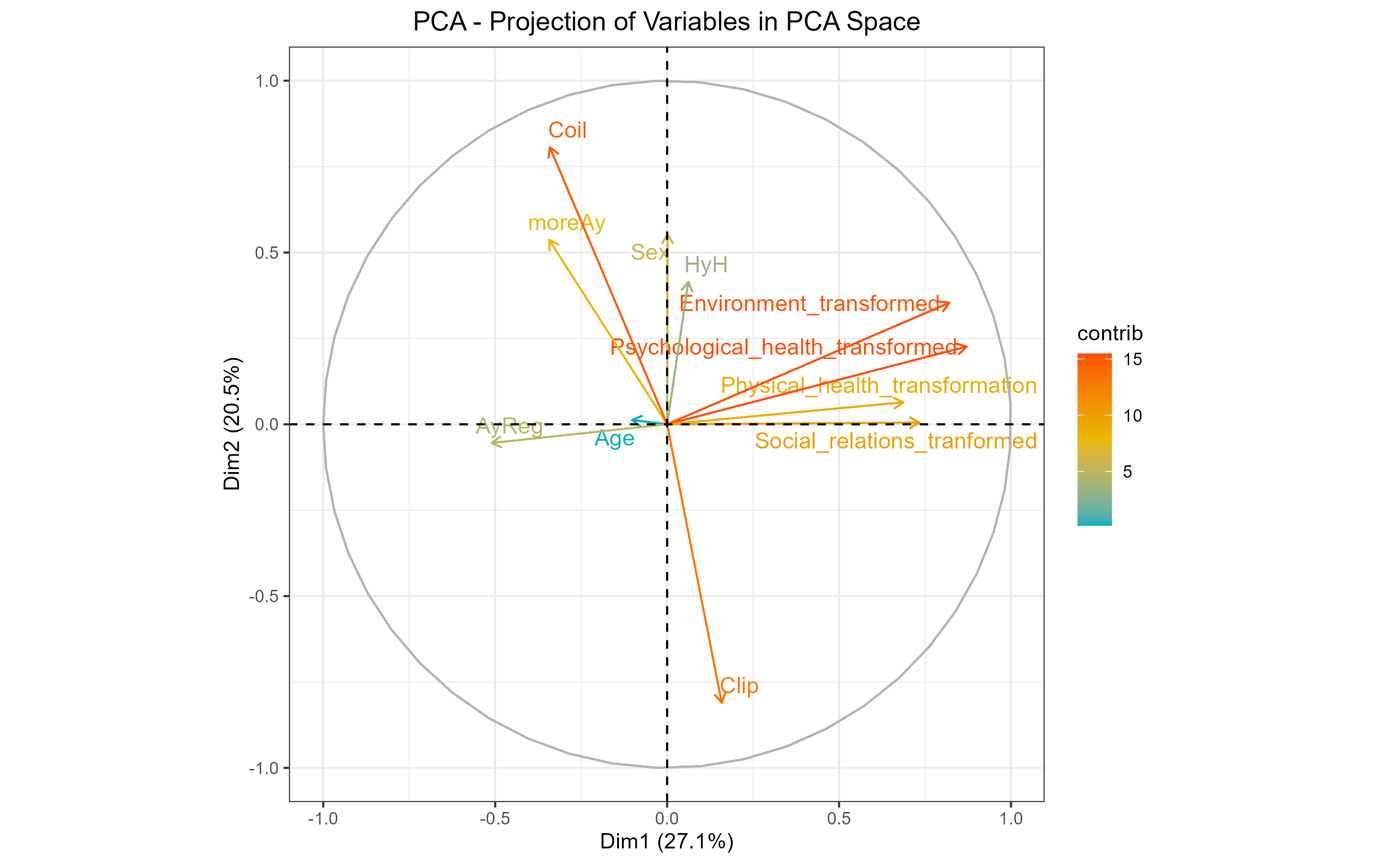


Suppl. Fig. 2. Graph of Components and Explained Variance (Left) and Biplot (Right) for Principal Component Analysis (PCA).

Supp. Table 2. Eigenvalues and variables used for Principal Component Analysis (PCA).

| Eigenvalues | | | | | | | |
| --- | --- | --- | --- | --- | --- | --- | --- |
|  | Dim 1 | Dim 2 | Dim 3 | Dim 4 | Dim 5 | Dim 6 | |
| Variance. | 3.232 | 2.304 | 1.938 | 1.509 | 1.142 | 0.713 | |
| % of var. | 24.859 | 17.723 | 14.905 | 11.607 | 8.783 | 5.483 | |
| Cumulative % of var. | 24.859 | 42.583 | 57.487 | 69.094 | 77.877 | 83.359 | |
| Variables | | | | | | | |
| Sex | -0.018 | 0.001 | 0.001 | 0.465 | 9.387 | 0.216 | |
| Age | -0.233 | 1.687 | 0.054 | -0.168 | -0.168 | 1.222 | |
| Psychological Health | 0.862 | 22.998 | 0.743 | 0.083 | 0.083 | 0.298 | |
| Social Relations | 0.666 | 13.712 | 0.443 | -0.146 | -0.416 | 0.923 | |
| Environment | 0.834 | 21.566 | 0.695 | 0.203 | 0.203 | 1.791 | |
| Physical Health | 0.772 | 18.347 | 0.593 | 0.055 | 0.055 | 0.132 | |
| University | 0.124 | 0.314 | 0.011 | 0.235 | 0.235 | 2.388 | |
| Job | 0.606 | 11.366 | 0.367 | 0.243 | 0.243 | 2.558 | |
| Clip | 0.072 | 0.163 | 0.005 | -0.831 | -0.831 | 29.909 | |
| Clip | -0.231 | 1.649 | 0.053 | 0.872 | 0.872 | 32.978 | |
| AyReg | -0.018 | 0.001 | 0.027 | 0.592 | 0.207 | 30.734 | |
| H&H | -0.233 | 0.046 | 0.265 | 0.367 | 0.058 | 4.487 | |
| MoreAy | 0.861 | 13.739 | 0.004 | 0.316 | -0.251 | 11.127 | |
| Individuals (3 first) | | | | | | | |
|  | Dist | Dim 1 | Ctr | Cos2 | Dim 2 | Ctr | Cos |
| 1 | 3.173 | -0.255 | 0.063 | 0.006 | 1.253 | 2.129 | 0.156 |
| 2 | 4.129 | 1.874 | 3.216 | 0.195 | 0.437 | 0.260 | 0.011 |
| 3 | 3.285 | 1.214 | 1.425 | 0.137 | -1.831 | 4.545 | 0.311 |
